# Perceptions of Important Characteristics of Physical Activity Facilities: Implications for Engagement in Walking, Moderate and Vigorous Physical Activity

**DOI:** 10.3389/fpubh.2017.00319

**Published:** 2017-11-28

**Authors:** Katie M. Heinrich, Christopher K. Haddock, Natinee Jitnarin, Joseph Hughey, LaVerne A. Berkel, Walker S. C. Poston

**Affiliations:** ^1^Functional Intensity Training Laboratory, Department of Kinesiology, Kansas State University, Manhattan, KS, United States; ^2^Institute for Biobehavioral Health Research, National Development and Research Institutes, Leawood, KS, United States; ^3^Department of Architecture, Urban Planning + Design, University of Missouri-Kansas City, Kansas City, MO, United States; ^4^Department of Counseling Psychology, University of Missouri-Kansas City, Kansas City, MO, United States

**Keywords:** built environment, physical activity, guidelines and recommendations, public health, recreation

## Abstract

**Background:**

Although few United States adults meet physical activity recommendations, those that do are more likely to access to physical activity facilities. Additionally, vigorous exercisers may be more likely to utilize a nearby physical activity facility, while light-to-moderate exercisers are less likely to do so. However, it is unclear what characteristics of those facilities are most important as well as how those characteristics are related to activity intensity.

**Purpose:**

This study examined relationships between self-reported leisure-time physical activities and the use of and perceived characteristics of physical activity facilities.

**Methods:**

Data were from a cross-sectional study in a major metropolitan area. Participants (*N* = 582; ages 18–74, mean age = 45 ± 14.7 years) were more likely to be female (69.9%), Caucasian (65.6%), married (51.7%), and have some college education (72.8%). Household surveys queried leisure-time physical activity, regular physical activity facility use, and importance ratings for key facility characteristics.

**Results:**

Leisure-time physical activity recommendations were met by 41.0% of participants and 50.9% regularly used a physical activity facility. Regular facility use was positively associated with meeting walking (*p* = 0.036), moderate (*p* < 0.001), and vigorous (*p* < 0.001) recommendations. Vigorous exercisers were more likely to use a gym/fitness center (*p* = 0.006) and to place higher importance on facility quality (*p* = 0.022), variety of physical activity options offered (*p* = 0.003), and availability of special equipment and resources (*p* = 0.01). The facility characteristics of low or free cost (*p* = 0.02) and offering childcare (*p* = 0.028) were barriers for walking, and being where friends and family like to go were barriers for moderate leisure-time physical activity (*p* = 0.013).

**Conclusion:**

Findings offer insights for structuring interventions using the social ecological model as well as for improving existing physical activity facilities.

## Introduction

Despite clear health benefits, the majority of the United States (US) population does not engage in the minimum recommended weekly levels of 150 min of moderate aerobic physical activity (including walking) and/or 75 min of vigorous aerobic physical activity in at least 10-min bouts (1). One quarter of the US population has reported no leisure time physical activity (LTPA) at all (2). Although 62% of US adults report meeting recommendations, only 9.6% of adults do so when objective accelerometer data are used (3). Men are more likely to meet aerobic activity recommendations than women (3, 4). In the Kansas City metropolitan area (the focus of the current study), just over 20% of adults report no LTPA (5), with only 48.6% of adults reporting meeting moderate or vigorous aerobic physical activity recommendations (6).

Individual and environmental barriers influence LTPA behaviors and include weather, lack of access to physical activity facilities, financial cost, and feeling tired (7). Specific barriers for walking include lack of time, family commitments, feeling tired, pollution, and costs (7). Work commitments are a key barrier for both moderate and vigorous LTPA, with financial cost inhibiting vigorous LTPA (7). Lack of access to and availability of physical activity facilities is linked to lower LTPA levels overall (8–10).

The social ecological model of physical activity posits that there must be a good “fit” between the person and their environment to facilitate physical activity (11). Environments may both facilitate and constrain different physical activity behaviors (11). Thus, selection of an appropriate physical activity facility for LTPA is crucial for behavior engagement, as *where* to exercise impacts the decision of *whether* to exercise (12). The compatibility between a person and a physical activity facility is termed recreation-environment fit and is optimized when one provides for the needs of the other, promoting LTPA behaviors (12). Individuals wanting to perform specific types of LTPA seek settings to support them (12). However, it is unclear whether it is the type of facility itself or more “microscale” characteristics within that determine initial and continued facility usage (13).

Limited research has detailed the different types of physical activity facilities utilized on a daily basis. In North Carolina, preferred locations for physical activity included streets and roads (41.7%), followed by homes (37.6%), private gyms (10.5%), worksite facilities (9.6%), and parks (8.6%) (14). In coastal Perth, WA, Australia, residents reported using streets (45.6%), public open spaces (48.8%), and beaches (22.3%) most frequently, although gyms/health clubs/exercise centers (10.8%), swimming pools (8.9%), sport/recreation centers (8.7%), and tennis courts (7.1%) were also used (15). Residents of Adelaide, SA, Australia reported using neighborhood streets, parks, homes, outdoor recreational settings, and health clubs/gyms most frequently (16).

Several types of objective audit instruments have been developed to assess physical activity facilities (13, 17, 18). One such instrument showed a relationship between fewer incivilities (e.g., litter) and increased walking, as well as greater accessibility and increased vigorous physical activity (19). However, to our knowledge, no audits have previously queried important characteristics of physical activity facilities from the user perspective.

Although the perceived characteristics of physical activity facilities that are most relevant for people engaging in LTPA in the US are unclear, a study in Greece found that the cleanliness of fitness centers, modern facilities (private facilities), and convenient transportation (public facilities) were most important for users (20). Key facility characteristics most likely also vary by the intended type of physical activity (e.g., walking, vigorous) as well as the demographic characteristics of the user (e.g., mothers may value childcare), although this has not been previously studied. It is possible that because of equipment needs for vigorous physical activity, use of specific physical activity facilities is necessary (21).

The primary aim of this study was to examine relationships between different types of physical activity facilities and self-reported LTPA of different intensities. We also examined how the perceived importance of various facility characteristics differed between those meeting and not meeting physical activity recommendations and by facility type.

## Materials and Methods

### Design and Setting

In this cross-sectional study, participant data were from the Kansas City Built Environment and Health Study (KC BEST) that used a three-group nested design in a five-county Midwest region from 2003 to 2009. Random selection of 21 census block groups (from >325) was made after stratification into low (<$23,386), middle ($23,386–$35,569), and high ($36,569.01–$150,001) income tertiles, with a minimum of 19% ethnic minority residents (22). In-person, door-to-door household surveys were completed in at least 25 homes per block group, for a total of 582 participants. Of the 1,928 adults contacted for participation; 30.2% were eligible and consented to participate.

### Participants

As shown in Table 1, participants completing the household survey were predominantly female (69.9%, *n* = 407), married (51.7%, *n* = 301), had some college education or more (72.8%, *n* = 424), and had annual household incomes of $60,000 or less (54.5%, *n* = 317). Participants’ ages ranged from 18 to 74, with an average age of 45 (SD = 14.7) years. Most participants were employed for wages (44.8%, *n* = 261), self-employed (13.9%, *n* = 81), or retired (13.7%, *n* = 80). Reported racial groups were similar to local population estimates (23) and included Caucasian (65.6%, *n* = 382), African American (25.1%, *n* = 146), Asian (2.6%, *n* = 15), American Indian (2.1%, *n* = 12), Hawaiian or Pacific Islander (0.5%, *n* = 3), or other (4.1%, *n* = 24); 6.2% (*n* = 36) were of Hispanic origin. On average, participants had lived at their present address an average of 10.6 (SD = 11.6) years.

**Table 1:** Participant characteristics (*N* = 582).

Variable	*N*	%	Mean (SD)	Range
Percent female	407	69.9		
Marital status				
Married	301	51.7		
Never married	111	19.1		
Divorced	86	14.8		
Widowed	29	5.0		
Member of an unmarried couple	26	4.5		
Separated	17	2.9		
Missing	12	2.1		
Education level				
Elementary (grades 1–8)	3	0.5		
Some high school (grades 9–11)	29	5.0		
High school graduate (grade 12 or GED)	114	19.6		
Some college or technical school	188	32.3		
College graduate	236	40.5		
Missing	12	2.1		
Household income (USD)				
≤$15,000	60	10.3		
$15,001–30,000	88	15.1		
$30,001–60,000	169	29.0		
$60,001–100,000	137	23.5		
$100,001–200,000	66	11.3		
>$200,000	15	2.6		
Missing	47	8.1		
Age (years)			45.2 (14.7)	18–74
18–20	15	2.6		
21–30	107	18.4		
31–40	116	19.9		
41–50	128	22.0		
51–60	113	19.4		
61–70	71	12.2		
71–74	32	5.5		
Employment status				
Employed for wages	261	44.8		
Self-employed	81	13.9		
Retired	80	13.7		
Homemaker	74	12.7		
Student	30	5.2		
Unemployed	25	4.3		
Unable to work	18	3.1		
Missing	13	2.2		
Race				
White	382	65.6		
Black	146	25.1		
Asian	15	2.6		
American Indian	12	2.1		
Hawaiian or Pacific Islander	3	0.5		
Other	24	4.1		
Ethnicity—percent of Hispanic origin	36	6.2		
Years living at present address			10.6 (11.6)	0–65

### Measures

#### Demographics

Age, sex, race/ethnicity, education, marital status, income, and employment status were assessed using questions derived from the Behavioral Risk Factor Surveillance System (24) and the US Census.

#### Self-Reported Physical Activity

Leisure time physical activity was determined using the International Physical Activity Questionnaire, which has been found to have acceptable reliability and validity (25). In brief, the questionnaire asked about the time spent being physically active during the past 7 days for recreation, sport, exercise, or leisure. Participants were asked to report the days and minutes they spent walking, doing vigorous activities (i.e., those that took hard physical effort), and doing moderate activities (i.e., those that took moderate physical effort). Responses were categorized into meeting walking or moderate physical activity recommendations (i.e., ≥150 min/week) and meeting vigorous physical activity recommendations (i.e., ≥75 min/week) (1).

#### Perceived Physical Activity Facilities and Characteristics

Participants were asked if they had a place where they usually worked out or did physical activities like exercise. Those who answered “yes” were subsequently asked to identify the facility type (i.e., park, gym/fitness center, school, church, community center, home/residence, other). Participants also were asked to rate the importance of 10 reasons for doing physical activity at that facility (e.g., it is close to where I live, there is a large assortment of physical activity options to choose from) on a scale from 1 “not at all important” to 7 “the most important.”

### Procedures

Teams of two trained data collectors approached randomly selected households in each block group to recruit participants. Since the overall survey asked a broad range of questions about not only physical activity but also food shopping and consumption, interviews were conducted with the person primarily responsible for making household food decisions. Individuals were eligible to participate if they had lived in their current neighborhood for at least 1 year, were between 18 and 74 years of age, did not have a health condition or disability that prevented them from participating in physical activity or exercise, felt comfortable reading and understanding a survey presented entirely in English, did not have a pacemaker or other electronic device in their body, and were not pregnant. After establishing eligibility, data collectors obtained written informed consent and then conducted a 60-min survey. Participants received a $20 gift card. All procedures were approved by the University of Missouri-Kansas City Institutional Review Board.

### Statistical Analysis

Descriptive statistics were used to summarize participants’ LTPA, as well as the frequency distribution and characteristics of regularly used physical activity facilities using IBM SPSS 24 (Armonk, NY, USA). Chi-square analysis was used to compare meeting each physical activity recommendation by gender and independent samples *t*-tests were used to compare meeting each recommendation by age. Chi-square analysis was used to examine use of a regular physical activity facility for those who met and did not meet walking, moderate or vigorous physical activity recommendations. To examine associations among physical activity facilities, perceived facility characteristics, and self-reported LTPA, mixed models were created within the SAS PROC GLIMMIX (SAS 9.3, SAS Institute Inc., Cary, NC, USA) with neighborhood as a random effect in each model to adjust for the sampling approach used in the study. Independent samples *t*-tests were used to examine gender differences as well as differences between individuals meeting versus not meeting each type of physical activity (i.e., walking, moderate, or vigorous) as independent variables and the mean importance rating for each perceived facility characteristic as the dependent variables. One-way ANOVA with Scheffe *post hoc* tests was used to examine differences in importance ratings for each perceived facility characteristic by age group. The *p*-value for all analyses was set at <0.05.

## Results

### Percent Meeting LTPA Recommendations by Category

In their leisure-time, 236 participants (40.5%) reported meeting one or more of the physical activity recommendation categories: walking 19.2% (*n* = 112), moderate 9.1% (*n* = 53), and vigorous LTPA 24.7% (*n* = 144); with 11.3% (*n* = 66) meeting more than one. Over half (56.4%, *n* = 328) did not meet the aerobic physical activity recommendations, including 26.1% (*n* = 152) who reported no LTPA in the past 7 days and 3.1% (*n* = 18) who did not provide LTPA data. Significantly more males than females met recommendations for moderate (15.8 versus 6.6%, χ^2^ = 11.99, *p* = 0.001) and vigorous LTPA (32.7 versus 22.3%, χ*^2^* = 6.90, *p* = 0.009).

Participants meeting moderate LTPA recommendations were significantly younger than those who did not [M = 38.1, SD = 13.9 years versus M = 45.6, SD = 14.5 years; *t*(565) = 3.62, *p* < 0.001], as were participants meeting vigorous LTPA recommendations [M = 41.3, SD = 14.1 years versus M = 46.2, SD = 14.6 years, respectively; *t*(564) = 3.48, *p* = 0.001].

### Types of Physical Activity Facilities Used by Participants

Use of a regular physical activity facility was reported by 50.9% of the sample (*n* = 296) and did not differ by gender. However, those who reported regular use of a physical activity facility were significantly younger (M = 43.8, SD = 14.6 years) than those who did not regularly use one [M = 46.3, SD = 14.6 years; *t*(571) = 1.99, *p* = 0.047]. Reported types of regularly used physical activity facilities included homes/residences (22.2%, *n* = 129), gyms/fitness centers (19.9%, *n* = 116), parks (2.9%, *n* = 17), community centers (2.6%, *n* = 15), or others (3.3%, *n* = 19; e.g., church, school, neighborhood, hospital, mall). Community centers and other facilities were collapsed into a composite “other” leaving three main categories for analysis: (1) homes/residences, (2) gyms/fitness centers, and (3) other community facilities.

### Associations between Type of LTPA and Type of Facility Used

Participants meeting recommendations for walking were significantly more likely to report having a regular facility in which to work out than those not meeting walking recommendations (23.1 versus 16.1%, χ^2^ = 4.391, *p* = 0.036). This also was true for participants who reported meeting recommended levels of moderate (13.3 versus 5.1%, χ^2^ = 11.06, *p* = 0.001) and vigorous LTPA (44.0 versus 5.5%, χ^2^ = 110.62, *p* < 0.001) as compared to those not meeting recommendations for each.

Models examining walking and moderate LTPA by type of physical activity facility were not significant (*p* > 0.05; data not shown). However, statistically significant differences were found for those meeting vigorous physical activity recommendations by type of physical activity facility (*F* = 3.93, *p* = 0.021), where participants who met vigorous physical activity recommendations were significantly more likely to exercise at a gym/fitness center than a home/residence (OR = 0.73, *p* = 0.006).

### Perceptions of Important Physical Activity Facility Characteristics

As shown in Table 2, the five most important facility characteristics reported for those who met physical activity recommendations and also reported regularly using a facility included being “close to where I live” (M = 6.0, SD = 1.7), “convenient to other places I often go” (M = 5.6, SD = 2.1), “affords me privacy or time alone” (M = 5.5, SD = 2.1), “free or less expensive to do physical activity there” (M = 5.3, SD = 2.1), and “place is of very high quality” (M = 5.2, SD = 2.1), with 31–64% of the sample rating those characteristics as 7 “the most important.”

**Table 2:** Average importance ratings for facility characteristics.

Characteristic	Average importance*^a^*; M (SD)	% ranking item as 7 (*n*)	Men; M (SD)	Women; M (SD)	Met walking recommendations; M (SD)	Met moderate PA^b^ recommendations; M (SD)	Met vigorous PA recommendations; M (SD)
Close to where I live (*n* = 296)	6.0 (1.7)	63.5 (188)	5.9 (1.8)	6.1 (1.7)	6.1 (1.7)	5.6 (2.1)	5.9 (1.8)
Convenient to other places I often go (*n* = 296)	5.6 (2.1)	45.3 (134)	5.4 (2.3)	5.7 (2.1)	5.7 (2.0)	5.5 (2.4)	5.5 (2.2)
Affords me privacy or time alone (*n* = 296)	5.5 (2.1)	47.3 (140)	5.3 (2.2)	5.6 (2.1)	5.6 (2.0)	5.2 (2.2)	5.3 (2.2)
Free or less expensive to do PA there (*n* = 296)	5.3 (2.1)	41.2 (122)	5.1 (2.0)	5.4 (2.1)	4.8 (2.2)^c^	5.0 (2.2)	5.2 (2.1)
Place is of very high quality (*n* = 295)	5.2 (2.1)	30.8 (91)	5.1 (2.0)	5.3 (2.1)	5.2 (2.0)	5.0 (2.0)	5.5 (1.9)^e^
Close to where I work^f^ (*n* = 296)	4.9 (2.8)	26.4 (78)	4.8 (2.8)	5.0 (2.8)	5.1 (2.8)	5.1 (2.8)	5.1 (2.7)
Large assortment of PA options (*n* = 294)	4.8 (2.2)	26.2 (77)	4.9 (2.1)	4.7 (2.3)	4.7 (2.2)	5.1 (2.3)	5.2 (2.1)^e^
Where my friends or family like to go (*n* = 296)	4.3 (2.7)	23.3 (69)	4.0 (2.6)	4.4 (2.7)	4.1 (2.7)	3.3 (2.6)^d^	4.1 (2.6)
Offers child care (*n* = 294)	4.9 (3.2)	22.1 (65)	4.9 (3.2)	5.0 (3.2)	4.2 (3.3)^c^	4.5 (3.3)	4.9 (3.2)
Special equipment/resources hard to find elsewhere (*n* = 295)	4.3 (2.5)	20.3 (60)	4.2 (2.6)	4.4 (2.5)	4.1 (2.7)	4.4 (2.3)	4.8 (2.4)^e^

*^a^Scale: 1, not important to 7, most important*.

*^b^Physical activity*.

*^c^Significantly lower rating than those not meeting walking recommendations (*p* < 0.05)*.

*^d^Significantly lower rating than those not meeting moderate PA recommendations (*p* < 0.05)*.

*^e^Significantly higher rating than those not meeting vigorous PA recommendations (*p* < 0.05–0.01)*.

*^f^Significantly higher rating for those ages 61–70 than those ages 21–30 (*p* = 0.032) significantly by age, ƒ(6,295) = 2.7, *p* = 0.015, with participants ages 61–70 years rating it significantly higher (M = 6.1, SD = 2.7) than those ages 21–30 years (M = 4.0, SD = 2.8; *p* = 0.032)*.

Importance ratings for perceived facility characteristics did not significantly differ by gender, but a few did vary significantly between participants who reported meeting and those who did meet recommendations (see Table 2). Those who failed to meet walking recommendations rated “free or less expensive to do physical activity there” and “it offers child care” significantly higher than those who met walking recommendations [*t*(292) = 2.34, *p* = 0.02 and *t*(290) = 2.22, *p* = 0.028, respectively]. Those who failed to meet moderate LTPA recommendations rated “where my friends or family like to go” significantly higher than those who met moderate recommendations, *t*(292) = 2.49, *p* = 0.013. However, participants who met vigorous LTPA recommendations had significantly higher ratings for the “place is of very high quality,” *t*(290) = −2.30, *p* = 0.022, there were a “large assortment of physical activity options,” *t*(289) = −3.01, *p* = 0.003, and there were “special equipment/resources that are hard to find elsewhere,” *t*(290) = −2.58, *p* = 0.01, than those who did not meet vigorous recommendations. Only “close to where I worked” differed.

Table 3 shows how each perceived characteristic was rated in importance by type of physical activity facility. Those who used a home/residence rated “close to where I live,” “affords me privacy or time alone,” and “offers child care” as significantly more important than those who used a gym/fitness center; they also rated “free or less expensive to do physical activity there” as significantly more important than both those who used a gym/fitness center or other community resource. Those who used a gym/fitness center rated “convenient to other places I often go,” “place is of very high quality,” “large assortment of physical activity options,” and “there is special equipment or resources that is hard to find elsewhere” as significantly more important than those who reported using a home or residence. Those who used an “other” type of community resource rated the “place is of very high quality” and has a “large assortment of physical activity options” as significantly more important than those using a home or residence.

**Table 3:** Average importance rating^a^ for facility characteristics by each participant’s primary type of facility (*n* = 296).

Characteristic	Home/residence, *n* = 129; M (SE)	Gym or fitness Center, *n* = 116; M (SE)	Other community facility, *n* = 19; M (SE)
Close to where I live	6.7 (0.2)^c^	5.4 (0.2)	5.7 (0.2)
Affords me privacy or time alone	4.7 (0.2)^c^	3.4 (0.2)	2.9 (0.4)
Convenient to other places I often go	3.8 (0.2)^c^	5.2 (0.2)	5.1 (0.3)
Free or less expensive to do PA^b^ there	5.7 (0.2)^d^	4.7 (0.2)	5.4 (0.3)
Place is of very high quality	4.5 (0.2)	5.5 (0.2)^e^	5.5 (0.3)^e^
Close to where I work	4.1 (0.3)	4.4 (0.3)	3.4 (0.4)
Large assortment of PA options	3.6 (0.2)	5.8 (0.2)^e^	4.8 (0.3)^e^
Where my friends of family like to go	4.0 (0.3)	3.7 (0.3)	4.3 (0.4)
Offers child care	6.7 (0.2)^c^	5.6 (0.2)	6.6 (0.5)
Special equipment/resources hard to find elsewhere	3.2 (0.2)	4.6 (0.2)^e^	4.1 (0.3)

*^a^Scale: 1, not important to 7, most important*.

*^b^Physical activity*.

*^c^Significantly greater than gym/fitness center (*p* < 0.05)*.

*^d^Significantly greater than gym/fitness center and other community (*p* < 0.05)*.

*^e^Significantly greater than home/residence (*p* < 0.05)*.

## Discussion

We examined relationships between LTPA, use of different types of physical activity facilities, and perceived importance of characteristics of those facilities. Less than half of our sample reported meeting physical activity guidelines, which was lower than national (3) but similar to other local data (6). We found that having a regular facility for physical activity was significantly related to meeting recommendations for each type of LTPA (i.e., walking, moderate, and vigorous), which was similar to previous research denoting the importance of facility access for meeting recommendations (8–10, 15, 26). We also found that significantly more men reported meeting both moderate and vigorous physical activity guidelines and that participants meeting moderate and vigorous guidelines were significantly younger than those who did not, similar to previous research (3, 4).

Study findings provide insight about the “fit” between our participants and important characteristics of their regular physical activity facilities, helping provide more microscale details to inform interventions framed by the social ecological model (11). We identified important characteristics that both facilitated vigorous physical activity and constrained moderate physical activity and walking, which have important public health implications (11). We found that participants meeting vigorous physical activity recommendations clearly valued having a high quality facility with a variety of exercise options and specialized equipment than those not doing enough vigorous activity, which may help explain why those who report exercising vigorously more often report using a facility (versus a home or park) to exercise (16). Conversely, for walking cost and child care were potential barriers for those not meeting recommendations, similar to previous research in Australia (7). Socializing with friends and family members was a potential barrier for those not meeting moderate physical activity recommendations, which differed from previous research that identified work commitments as a key barrier (7) as well as research showing longer moderate exercise bouts with friends and family members (27). Significant differences in importance of several facility characteristics were found between participants who met or did not meet physical activity recommendations and facility type, but not by gender. For those meeting physical activity recommendations the most important characteristics reflected proximity to home, convenience, time alone, low/free cost, and quality. These factors addressed some of the most common barriers to physical activity including access, time, and cost (7–10).

Although gym/fitness center use was 2% less than home/residence use in our study, fitness facilities and health clubs are increasingly being used by Americans, with 29,750 health clubs serving 45.3 million members in 2009 [in 2016, 36,540 health clubs serve 57.3 million members (28)]. For those doing vigorous physical activity, gyms/fitness centers were used significantly more than homes/residences, as hypothesized by Sallis and associates (21), although other research has found both gym and home use equivocal for vigorous exercise bouts (27). Highest rated facility characteristics for vigorous physical activity included proximity/accessibility, convenient location, quality, privacy or time alone, and low/free cost. Previous research also found people liked to spend time alone during vigorous exercise (27).

We found that those who primarily used a home or residence saw proximity/accessibility, privacy, childcare, and low cost as most important, adding to the literature which is replete with research on exercise at home for clinical and older adult populations [e.g., Ref. (29–31)], but not healthy adults. Gym or fitness center users prioritized location (near to other frequented places), quality, variety of exercise options, and specialized equipment as most important. Those using other community resources had higher ratings for having a variety of exercise options. Use of homes/residences and other community resources such as parks as well as use of gyms or fitness centers was similar to previous research in the US and Australia (14–16).

Although not the focus of our study, low income individuals face significant barriers to exercise when public facilities charge for admission or class participation; significant barriers to class participation include cost, childcare, not wanting to attend alone, lack of time, and health issues (32). As individuals with disabilities are disproportionately represented among the poor and undereducated US population (33), a recent finding that many fitness facilities across the US were inaccessible and failed to provide individuals with disabilities an equitable opportunity for exercise is concerning (34). As well, research in the St. Louis, MO, USA area found that odds of running and walking routes reported in high-socioeconomic status (SES) neighborhoods were significantly greater than those for low-SES neighborhoods, with 52% lower odds of running and 64% lower odds of walking in a park in the low-SES neighborhoods despite similar numbers of available parks (35). Additionally, the overall odds of running and walking in low-SES neighborhoods was significantly lower than in high-SES neighborhoods, highlighting environmental health disparities in the area (35).

Study strengths include random selection of both neighborhoods and participants within those neighborhoods, comprising a wide range of SES groups in a large metropolitan area. The study population was representative of the metropolitan area as a whole (23). As well, the IPAQ has been shown to have acceptable test–retest reliability for determining participants who meet recommendations as well as moderate reliability for overall minutes of activity (36) and is an acceptable measure for assessing population physical activity levels among adults in diverse settings (25).

A limitation of our study was that we did not have an objective measure of physical activity to verify actual levels of LTPA (3). Questions used for use of and importance ratings for physical activity facilities were created for the study and lack reliability and validity analysis. Due to the cross-sectional nature of the study, we were unable to determine the direction of influence for the recreation-environment fit for participants (12) to know whether individuals sought settings to support specific types of LTPA or whether their facility use shaped the type of physical activity in which they ultimately engaged (13). Future research could use a longitudinal design in order to determine direction of relationships. Future research might also query the number of available physical activity facilities in each participant’s neighborhood, which may have contributed to differences in meeting recommendations (8–10), as well as include objective audits of the facilities themselves (13). It would also be helpful to further differentiate between sub-types of moderate and vigorous activities (e.g., weight training, jogging, aerobics, and high-intensity exercise) and facility use.

## Conclusion

This study provides necessary contemporary data about the type of exercise performed in relation to the type of physical activity resource utilized. We identified key perceived characteristics of physical activity facilities along with types of facilities that were most important for adults who met walking as well as moderate and vigorous physical activity recommendations. These data should be useful for structuring physical activity interventions utilizing the social ecological model as well as for improving existing facilities for physical activity in diverse metropolitan areas. At individual or household levels, these findings imply that increasing access to physical activity facilities and thus meeting recommended activity levels may be a matter of removing barriers to and providing resources for in-home exercise. In neighborhood or community environments, increasing the closeness or functional proximity of high-quality, high-variety physical activity facilities may be key for facilitating increased LTPA, especially vigorous intensity.

## Ethics Statement

All procedures were approved by the University of Missouri-Kansas City Institutional Review Board. After establishing eligibility, data collectors obtained written informed consent and then conducted a 60-min survey. Participants received a $20 gift card.

## Author Contributions

KH helped to collect and analyze study data, conceived the manuscript idea, and led the manuscript writing. CH helped to design the study, collect and analyze study data, and assisted with manuscript writing. NJ helped to collect and analyze study data and assisted with manuscript writing. JH helped to design the study, collect data, and assisted with manuscript writing. LB helped to collect study data and assisted with manuscript writing. WP designed the study, collected and analyzed study data, and assisted with manuscript writing.

## Conflict of Interest Statement

The authors declare that the research was conducted in the absence of any commercial or financial relationships that could be construed as a potential conflict of interest.
